# A comparison of the cytotoxic potential of standardized aqueous and ethanolic extracts of a polyherbal mixture comprised of *Nigella sativa* (seeds), *Hemidesmus indicus* (roots) and *Smilax glabra* (rhizome)

**DOI:** 10.4103/0974-8490.75451

**Published:** 2010

**Authors:** Sameera R. Samarakoon, Ira Thabrew, Prasanna B. Galhena, Dilip De Silva, Kamani H. Tennekoon

**Affiliations:** *Institute of Biochemistry, Molecular Biology and Biotechnology, University of Colombo, Cumarathunga Munidasa Mawatha, 90, Colombo 3, Sri Lanka*; 1 *Department of Biochemistry and Clinical Chemistry, Faculty of Medicine, University of Kelaniya, Talagoole Road, Ragama, Sri Lanka*; 2 *Department of Chemistry, Faculty of Science, University of Colombo, Sri Lanka*

**Keywords:** Cytotoxicity, *Hemidesmus indicus*, MTT and SRB assays, *Nigella sativa*, *Smilax glabra*, Standardization

## Abstract

**Background:**

A decoction (hot-water extract) comprised of *Nigella sativa* (seeds), *Hemidesmus indicus* (roots), and *Smilax glabra* (rhizome) has been reported to prevent chemically-induced hepatocarcinogenic changes in rats and to exert significant cytotoxic effects on human hepatoma (HepG2) cells. However, the decoction used in previous studies to determine cytotoxicity was not standardized. Further, during preparation of pharmaceuticals for clinical use, it is more convenient to use an ethanolic extract. Therefore this study was carried out to (a) develop standardized aqueous and ethanolic extracts of the plant mixture (*N. sativa, H. indicus,* and *S. glabra*) used in the preparation of the original decoction, and (b) compare the cytotoxic effects of these two extracts by evaluating cytotoxicity to the human hepatoma (HepG2) cell line.

**Methods:**

Aqueous and ethanolic extracts have been standardized by evaluating organoleptic characters, physicochemical properties, qualitative and quantitative analysis of chemical constituents, and analysis of High Performance Liquid Chromatography (HPLC) and Thin Layer Chromatography (TLC) profiles. Cytotoxic potentials of the above standardized extracts were compared by evaluating their effects on the survival and overall cell activity of HepG2 cells by use of the 3-(4, 5-dimethylthiazol-2yl) -2, 5 – biphenyl tetrazolium bromide (MTT) and Sulphorhodamine B (SRB) assays.

**Results:**

Results from MTT and SRB assays demonstrated that both extracts exerted strong dose-dependent *in vitro* cytotoxicity to HepG2 cells. The standardized aqueous extract showed a marginally (though significantly, *P*<0.05) higher cyotoxic potential than the ethanolic extract. Thymoquinone, an already known cytotoxic compound isolated from *N. sativa* seeds was only observed in the standardized ethanolic extract. Thus, compounds other than thymoquinone appear to mediate the cytotoxicity of the standardized aqueous extract of this poly-herbal preparation.

**Conclusion:**

It may be concluded that results obtained in the present study could be used as a diagnostic tool for the correct identification of these aqueous or ethanolic extracts and would be useful for the preparation of a standardized pharmaceutical product that may be used in the future for clinical therapy of hepatocellular carcinoma.

## INTRODUCTION

Cancer is an important public health concern and is considered to be the second largest common disease in the world.[1] Hepatocellular carcinoma is the most frequent primary liver cancer, fifth commonest neoplasm in the world, and third commonest cause of cancer-related death. More than 500,000 new cases are diagnosed annually worldwide and are most common in men in many developing countries.[2–4] Hepatocellular carcinoma is very common in the Asian region and it is strongly associated with chronic liver infection or hepatitis, especially Hepatitis B and C viruses. Other important risk factors include liver cirrhosis from excessive alcohol consumption as well as ingestion of aflatoxin, a substance which is found in moldy nuts and grain.[4,5]

Chemoprevention, the prevention of cancer by ingestion of chemical agents that reduce the risk of carcinogenesis, is one of the most direct ways to reduce the morbidity and mortality of cancer.[5] Plants have been a prime source of highly effective conventional drugs for the treatment of many forms of cancer. It is estimated that approximately 60% of most effective antitumor/ anti-infectious drugs already on the market or under clinical investigations are of products or compounds derived from natural products.[6–9]

In Asian countries poly-herbal preparations are often used by traditional medical practitioners for the treatment of cancer.[10] One such herbal remedy prescribed to cancer patients by a family of indigenous medical practitioners in Sri Lanka, is a decoction (hot-water extract) prepared from *Nigella sativa* (seeds), *Hemidesmus indicus* (roots) and *Smilax glabra* (rhizome) (personal communication, Ayurvedic physician, Dr, Nimal Jayathilaka). In previous investigations, Iddamaldeniya *et al*.,[11,12] have demonstrated that protection against diethylnitrosoamine (DEN)-mediated carcinogenic changers in rat liver can be achieved by long-term treatment with this decoction. Recent investigations by Thabrew *et al*.,[10] have also shown that the decoction can exert a significant dose-dependent cytotoxic effect on human hepatoma (HepG2) cells. However, the decoction used in these investigations was not properly standardized. Standardization is an essential measurement for ensuring the quality control of herbal drugs.[13] To develop a herbal extract with a pharmaceutical product that can be used in modern clinical practice, pharmaceutical companies prefer to use an alchoholic extract as the starting material than an aqueous extract. Therefore the present investigation was carried out to (a) develop standardized aqueous (hot-water extract) and ethanolic extracts of the herbal mixture previously utilized in the evaluation of antihepatocarcinogenic[11,12] and cytotoxic activities,[10] and (b) compare the cytotoxic potential of the above standardized extracts by evaluating their effects on the survival and overall cell activity of HepG2 cells by use of MTT and SRB assays.

## MATERIALS AND METHODS

### Collection of plant materials

Plant material for the preparation of extracts was purchased from a reputed vendor of herbal material used by traditional medical practitioners in Sri Lanka (D. J. Fenando Pvt Ltd, Gabose lane, Colombo 13). The identities were confirmed by the Botanist at Bandaranayaka, Memorial Ayurvedic Research Institute (BMARI), Navinna, Maharagam Sri Lanka. Voucher specimens of *N. sativa* (seeds), *H. indicus* (roots), and *S. glabra* (rhizome) have been deposited in the Institute of Biochemistry, Molecular biology and Biotechnology, University of Colombo, Sri Lanka. (Voucher specimen nos.UOC/IBMBB/2009/01, UOC/IBMBB/2009/02 and UOC/IBMBB/2009/03).

### Standardization of the aqueous and ethanolic extracts

Aqueous (hot-water) and ethanolic extracts of 15 different samples of plant materials purchased at different times of the year from the same vendor were standardized according to the methods recommended by the World Health Organization (WHO).[14] Estimation of organoleptic characters, physicochemical properties, qualitative and quantitative analysis of chemical constituents, and analysis of HPLC, and TLC profiles, were carried out.

### Preparation of aqueous extract (hot-water extract)

Sixty grams (60 g) of plant material (composed of a mixture of 20 g each of *N. sativa* seeds, *H. indicus* roots and *S. glabra* rhizomes) was ground and boiled gently with 1.6 L distilled water for approximately 3 h to reduce the volume to 200 ml. The extract was then filtered through a layer of muslin, filtrate centrifuged at 3000 g for 15 min to remove any debris, and the supernatant freeze dried and stored at -20°C until required.

### Preparation of ethanolic extract

The dried, powdered plant material mixture (60g) was subjected to soxhlet extraction with 80% ethanol (500 ml), filtered, and evaporated to dryness under reduced pressure, stored at -20°C until required.

### Determination of Physicochemical parameters

#### Determination of pH

The pHs of the aqueous and ethanolic extracts were determined using a pH meter (Fisher brand Hydrus 300, USA), at room temperature.

#### Determination of water and ethanol-extractable matter in the mixture of air-dried N. sativa seeds, H. indicus roots and S. glabra rhizomes

Air-dried *N. sativa* seeds, *H. indicus* roots *and S. glabra* rhizomes were mixed in equal proportions (3 g each) and coarsely powdered; 4g of the above mixture was placed in an accurately weighed glass-stoppered conical flask. For estimation of water-extractable matter, distilled water (100 ml) was added to the flask and weighed to obtain the total weight including the flask. The contents were shaken well and allowed to stand for 1 h. A reflux condenser was attached to the flask and boiled gently for 1 h, cooled and weighed. The flask was readjusted to the original weight with distilled water. The mixture was shaken well and filtered rapidly through a dry filter. Then 25 ml of the filtrate was transferred to a round-bottomed flask and evaporated to dryness on a water bath. Finally, it was dried at 105°C for 6 h, cooled in a desiccator for 30 min, and immediately weighed. Same procedure was followed using ethanol instead of distilled water to determine extractable matter in ethanol. The extractable matter was calculated as mg/g of air-dried material.[14]

#### Determination of total ash content of extracts

Two grams of dried extract was placed in a crucible and weighed. Dried material was spread in an even layer in the crucible, and the material ignited by gradually increasing the heat to 500-600°C until free from carbon, cooled in a desiccator, and weighed. Total ash content was calculated in mg/g of dried extract.[14]

#### Determination of acid insoluble ash

Twenty-five ml of 2M HCl was added to the crucible containing the total ash, covered with a watch glass and boiled gently for 5 min. The watch glass was rinsed with 5 ml of hot water and this liquid added to the crucible. The insoluble matter was collected on an ashless filter paper and washed with hot water until the filtrate was neutral. The filter paper containing the insoluble matter was transferred to the original crucible, dried on a hot plate and ignited to constant weight. Acid-insoluble ash content was calculated as mg/g of dried extract.[14]

#### Determination of water-soluble ash

Twenty-five ml of water was added to the crucible containing the total ash and boiled for 5 min. Insoluble matter was collected on an ashless filter paper, washed with hot water and ignited for 15 min at a temperature not exceeding 450°C. The weight of the residue was subtracted from the total ash and the water-soluble ash content calculated as mg/g of dried extract.[14]

#### Determination of foaming index

The dried water extract (1 g) was dissolved in 100 ml water, the solution cooled and filtered. The ethanolic extract (1 g) was dissolved in 2.5 ml 95% ethanol, the final volume made up to 100 ml with distilled water, the solution cooled and filtered. Each of the above filtrates were placed in test tubes in a series of successive portions of 1,2,3 up to 10 ml and the volumes in each tube adjusted with distilled water to 10 ml. The tubes were stoppered and then shaken for 15 sec in a lengthwise motion (2 frequencies / sec). After allowing the tubes to stand for 15 min, the height of foam was measured by means of a graduated tape with millimeter scale.[14]

### Qualitative and Quantitative Analysis of chemical constituents

#### Preliminary phytochemical screening of aqueous and ethanolic extracts

The preliminary phytochemical screening of the aqueous and ethanolic extracts was carried out using standard laboratory procedures, to detect the presence of different secondary metabolites such as alkaloids, flavonoids, saponins, tannins, steroids, reducing sugars, and phenols.[15–17]

#### Determination of polyphenolic content

The total polyphenolic content was estimated according to the Folin-Ciacalteau method described by Spanos and Worlstad.[18] The freeze-dried aqueous extracts were re-dissolved in distilled water, filtered, and the concentrations of each filtrate adjusted to 10 mg/ml with distilled water. Each ethanolic extract was re-dissolved in 95% methanol to a concentration of 10 mg/ml. Each aqueous extract and ethanolic extract (0.05 ml) was diluted with distilled water or 80% methanol (0.95 ml) respectively and mixed with 5 ml of 10-fold diluted solution of 2N Folin-Ciocalteau reagent (Sigma-UK). Four milliliters of saturated sodium carbonate solution were then added to the mixture and shaken. After incubation at room temperature for 2 h the absorbance of the reaction mixture was measured at 760 nm against a methanol blank. Gallic acid (Sigma-Aldrich Chemie, Steinheim Germany) (0-100 mg/l) was used as a standard to prepare a calibration curve. The total phenolic content was expressed in mg of gallic equivalents (GAE)/100 g of extract.

#### Determination of total flavonoid content

Dried aqueous and ethanolic extracts were re-dissolved in 95% methanol to a final concentration of 10 mg / ml. The total flavonoid content was determined using the Dowd method as adapted by Meda *et al*.[19] Briefly, 5 ml of 2% aluminium trichloride (AlCl_3_) in methanol was mixed with 1 ml of extract (10 mg/ml). Absorbance readings at 415 nm were taken after 10 min against a blank sample consisting of 5-ml extract plus 5 ml methanol without AlCl_3_. The total flavonoid content was determined using a standard curve prepared with quercetin (Sigma-Aldrich Chemie, Steinheim Germany) (0-30 mg/l) as the standard. The total flavanoid contents are expressed as mg of quercetin equivalent (QE)/100 g of extract.

#### Determination of heavy metal composition

Samples of (0.5 g) dried aqueous extracts and ethanolic extracts of the mixture of plants comprised of *N. sativa, H. indicus,* and *S. glabra*, were placed in clean silica crucibles, digested with a mixture of acids having a ratio of conc. HNO_3_ to HClO_4_ acid 1:1 and heated up to 135°C until a white residue was obtained. The resulting dry inorganic residue in each crucible was dissolved in 20 ml of distilled water and used for the estimation of the heavy metal concentration.

The digested samples were analyzed for Cd and Pb, using a graphite furnace atomic absorption spectrophotometer (AAS; GBC 932 plus, Australia). A single-beam hollow cathode lamp (GBC) was used for estimation of Cd and Pb. Concentration of Hg was determined through AAS using Cold Vapor technique and air-acetylene flame was used for determination of As concentration. The metal quantification was based on calibration curves, which were determined through a series of concentrations prepared by using the chemical standard with 1000 mg/l concentration.[20] The concentration of the respective metals in samples were expressed as mg of metal per kg (ppm). The lowest detectable levels of Cd, Pb, As and Hg according to methods used were 0.004 mg /L, 0.1 mg /L, 2 mg /L and 1.5 mg /L respectively.

### Analysis of High-performance liquid chromatography (HPLC) and Thin layer chromatography (TLC) profiles

#### HPLC profiles

For determination of the HPLC profiles, the aqueous extracts and ethanolic extract were re-dissolved in distilled water, 80% methanol, respectively (20 mg/ml concentration). After filtration through 0.45 µm, 13-mm Millipore filters, 20 µl of each sample were injected into an Intertsil ODS-3 C_18_, (250 × 4.0 mm, 5 µm) reversed phase column of a High-Performance Liquid Chromatography System (Schimadzu, Kyoto, Japan) connected to a UV-Vis detector (Model SPD-10AVP). The HPLC analysis was performed using a linear gradient of 80% water in methanol to 100% methanol for 30 min, followed by100% methanol for 50 min with a flow rate of 0.5 ml / min, detection 254 nm. The peak areas and peak heights were analyzed by the software package (CLASS-VP) provided with the HPLC system. For determination of HPLC profile of thymoquinone, the chemical purchased from Sigma Aldrich, USA was re-dissolved in DMSO (1 mg/ml concentration) and 10 µl of the sample was injected into the HPLC system. HPLC analysis was performed as described above for the aqueous and ethanolic extracts. All solvents were HPLC grade and purchased from Fisher Scientific International Company, UK.

#### TLC profiles of aqueous and ethanolic extracts

Twenty microliters of each extract was spotted onto TLC plates coated with silica gel (pre-coated, GF_254_)and separated using a variety of solvent systems. The best separation of the aqueous extracts occurred in the solvent system comprised of Butanol: Acetic acid: water (60: 15: 25 v/ v) while the best solvent system for separation of the ethanolic extracts was found to be a mixture of Methanol: Cyclohexane: Dichloromethane (6: 20: 74 v/v). Vanillin sulphate spray reagent was used to visualize spots. All solvents were analytical grade and purchased from Fisher Scientific International Company, UK.

### Cytotoxicity assays

#### Cell culture

HepG2 (human hepatoma) cells were harvested by trypsinization, plated (5 × 10^3^ cells/well) in 96-well cell culture plate and maintained in Dulbecco’s Modified Eagle Medium (DMEM) for 24 h at 37°C in 95% air / 5% CO_2_ atmosphere, with 95% humidity. Cultures were exposed only to medium (1% DMSO, controls) or medium containing different concentrations of aqueous and ethanolic extracts dissolved in 1% DMSO (300 µg/ml -4800 µg/ml), and incubated for 24 h. At the end of this incubation period, cells were briefly washed with Phosphate-buffered saline (PBS). Fresh medium (200 µl) was then placed in each well and Sulphorhodamine (SRB) and MTT (3-(4,5-Dimethylthiazol-2-yl)-2,5-diphenyltetrazolium bromide) assays performed as described below.

#### Sulphorhodamine (SRB) cytotoxicity assay

The SRB cytotoxicity assay was performed according to the method of Mitry *et al*.[21] Cell survival was determined after exposure to different concentrations of the aqueous extracts or ethanolic extracts for 24 h. Cells were fixed with 50 µl of ice-cold 50% trichloroacetic acid solution by gently adding on top of the medium overlaying the cells. The plates were then incubated for 60 min at 4°C. Wells were rinsed five times with tap water and then cells were stained with 0.4% SRB solution (100 µl stain/well) for 15 min at room temperature. After staining, SRB solution was poured off, unbound dye was removed by washing five times with 1% acetic acid solution and left to air dry. The bound SRB dye was then solubilized by adding unbuffered Tris-base solution (200 µl/well), and plates were placed on a plate shaker for 1 h at room temperature. Plates were then read at OD 540 nm, using a microplate reader (EL_x_ 800 Universal Micro Plate Reader, BIO-TEK INSTRUMENTS, USA) and the results expressed as a percentage of control values.

#### Overall cell activity – MTT assay

Effect on overall cell activity was determined by performing the MTT assay based on the method of Oka *et al*.[22] The MTT assay measures the metabolism of 3-(4, 5-dimethylthiazol-2yl) -2, 5 – biphenyl tetrazolium bromide to form an insoluble formazan precipitate by mitochondrial dehydrogenases only present in viable cells. After exposure of cells to different concentrations of the aqueous extract or ethanolic extract for 24 h, twenty (20 µl) microlitres of MTT solution was added to the 200 µl medium in each well of the 96-well plate, and the plate was incubated at 37° C for 4 h. The medium was then removed by aspiration. Finally, 100 µl isopropanol / HCl was added per well, the plate was shaken for a further 30 min and the absorbance at 620 nm was measured using a microplate reader EL_x_ 800 Universal Microplate Reader, BIO-TEK INSTRUMENTS, USA) and the results expressed as a percentage of control values.

### Statistical analysis

Statistical analysis of the results obtained in each experiment was carried out by use of the MINITAB 14 statistical software package.

## RESULTS

### Physicochemical parameters

Physicochemical parameters of the aqueous and ethanolic extracts were determined according to the methods recommended by World Health Organization (WHO). As apparent from Table 1, the percentage yield of ethanolic extract and ethanol-extractable matter were greater than the percentage yield of aqueous extract and water-extractable matter. A higher content of total ash, acid-insoluble ash and water-soluble ash were found in the aqueous extract in comparison to the ethanolic extract. Analysis of heavy metals (Cd, Hg, Pb and As) showed the absence of detectable levels of these metals in both aqueous and ethanolic extracts.

**Table 1:** Physicochemical parameters

Parameter	Aqueous extract	Ethanolic extract
Yield (%) on dry wt. basis (Mean±SD)	15.242 ± 0.319	21.892 ± 0.231
Extractable matter (mg/g, Mean%±SD)	132.5 ± 2.260	178.5 ± 3.340
Total ash (Mean%±SD)	5.9068 ± 0.449	2.2131 ± 0.129
Acid-insoluble ash (Mean%±SD)	0.3791 ± 0.065	0.2174 ± 0.007
Water-soluble ash (Mean%±SD)	3.8832 ± 0.345	0.6155 ± 0.027
Foaming index	<100	<100
pH value of (Mean±SD)	4.86 ± 0.130	4.72 ± 0.070
Heavy metal content (ppm)		
Pb	ND	ND
Cd	ND	ND
As	ND	ND
Hg	ND	ND

Values are expressed as mean ± S.D., n = 15. ND = Not detected

### The organoleptic properties of aqueous and ethanolic extracts

As seen in Table 2, both the aqueous and ethanolic extracts had similar organoleptic properties except for the darker color of the aqueous extract.

**Table 2:** Organoleptic properties of aqueous and ethanolic extracts

Name of the extract	Appearance	Color	Taste	Smell
Aqueous extract	Liquid	Dark Brown	Astringent	Pungent
Ethanolic extract	Liquid	Brown	Astringent	Pungent

### Phytochemical analysis and total polyphenol and flavonoid contents of aqueous and ethanolic extracts

Phytochemical screening carried out using standard laboratory procedures demonstrated the presence of alkaloids, flavanoids, saponins, tannins, steroids, reducing sugars and phenolic compounds in both the aqueous and ethanolic extracts. Higher saponin content was found in the aqueous extract than in the ethanolic extract [Table 3]. As seen in Table 4, a greater content of total polyphenolic and flavanoid compounds were found in the ethanolic extract when compared to the aqueous extract.

**Table 3:** Phytochemical analysis of water extract and ethanolic extracts

Components	Water extract	Ethanolic extract
Alkaloids	+	+
Flavonoids	+++	+++
Saponins	+++	+
Tannins	+++	+++
Steroids	+++	+++
Reducing sugars	+++	+++
Phenols	+++	+++

+++ = appreciable amount; + = Trace amount

**Table 4:** Total polyphenol content and flavonoid content in aqueous and ethanolic extracts

Name of the extract	Total polyphenols (mg GAE/100g ±SD)	Total Flavonoids (mg QE/100g ±SD)
Aqueous extract	23.80 ± 4.563	4.566 ± 1.004
Ethanolic extract	69.40 ± 5.029	5.518 ± 1.022

Values are expressed as mean ±S.D., n = 15. Polyphenolic content is expressed as gallic acid equivalents (GAE) while total flavanoid contents are expressed as Quercetin equivalents (QE).

*TLC analysis of water extract and ethanolic extracts*.Table 5 summarizes the R_f_ values of spots visible in the TLC profiles of the aqueous and ethanolic extracts.

**Table 5:** TLC analysis of aqueous and ethanolic extracts

Name of the extract	Solvent system	Rf values of the spots
Hot-water extract	Butanol: Acetic acid: Water,	
	(60: 15: 25 v/ v)	0.055*
		0.417*
		0.484#
		0.747*
		0.857$
		0.923*
Ethanolic extract		
	Methanol: Cyclohexane:	0.033*
	Dichloromethane	0.080*
	(6: 20: 74 v/v)	0.127#
		0.273#
		0.320#
		0.360$
		0.380$
		0.460*
		0.633*
		0.753$
		0.990$

* - intense,

$ - Moderately intense,

# - Faint

### HPLC analysis of the aqueous and ethanolic extracts

**Figure 1:**
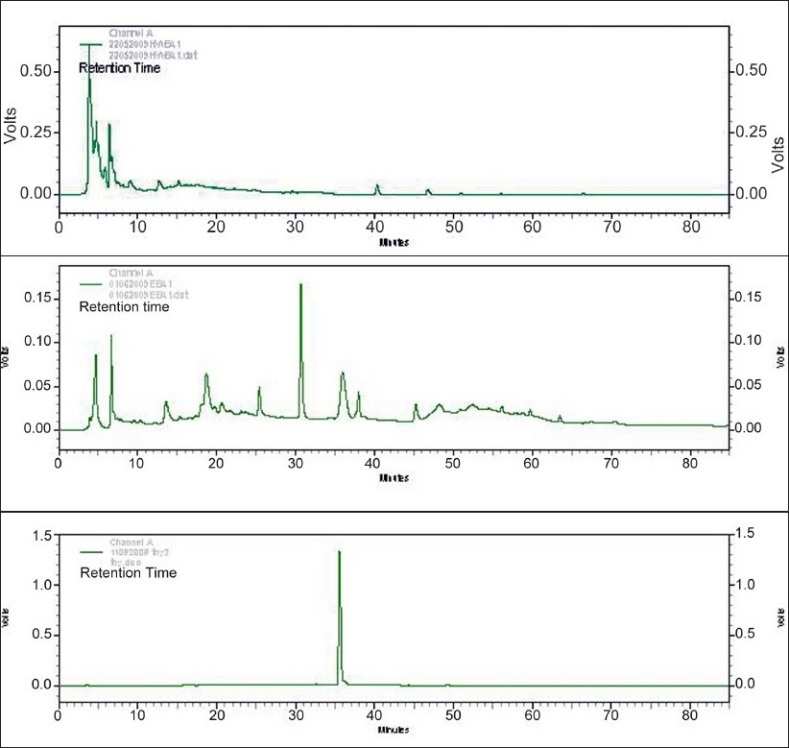
(a) HPLC profile of aqueous extract, (b): HPLC profile of ethanolic extract (c): HPLC profile of Thymoquinone

Each extract was subjected to reverse phase chromatography as described in the ‘Materials and Methods’ section. As apparent in Figures 1a and 1b, most of the major peaks in the aqueous extract appeared at retention times < 20 min while in the ethanolic extract, major peaks with retention times up to 40 min were also observed. This profile was observed in all 15 samples of each extract that was analyzed.

A comparison of the HPLC profiles of the aqueous and ethanolic extracts (Figures 1a and 1b) with that of thymoquinone (a cytotoxic component present in *N. sativa* seeds) showed (Figure 1c) that thymoquinone (retention time of 35.98 min) was only present in the ethanolic extracts and not in the aqueous extracts.

### Effect on overall cell viability-MTT assay

**Figure 2:**
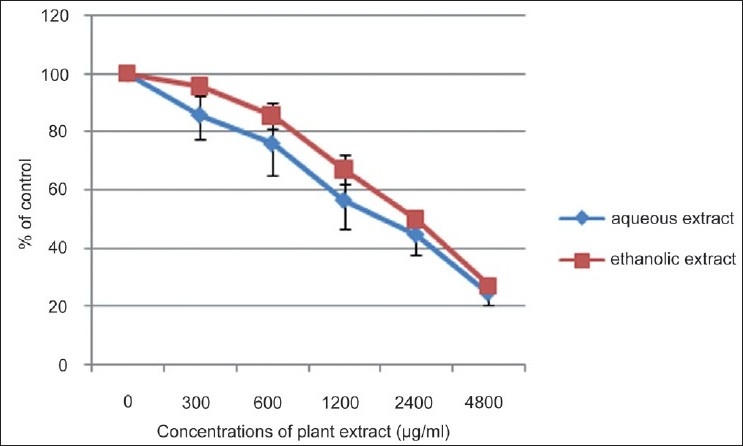
Effect of aqueous and ethanolic extracts on overall cell viability-MTT assay. Mean±S.D.

The effects of the aqueous and ethanolic extract on overall activity of HepG2 cells were tested by MTT assay [Figure 2]. Both the aqueous extract and ethanolic extracts demonstrated a dose-dependent reduction in the overall activity of HepG2 cells with the maximum effect at concentrations > 4800 µg/ml. The dose causing 50% inhibition, ED_50_ ‘s of 24-h post incubation periods of aqueous extract and ethanolic extract were 1600 µg/ml, and 2400 µg/ml respectively. The inhibition by aqueous extract was marginally (though significantly) greater (*P*< 0.05) than that mediated by the ethanolic extract at all the different concentrations tested.

### Relative cell survival-SRB assay

**Figure 3:**
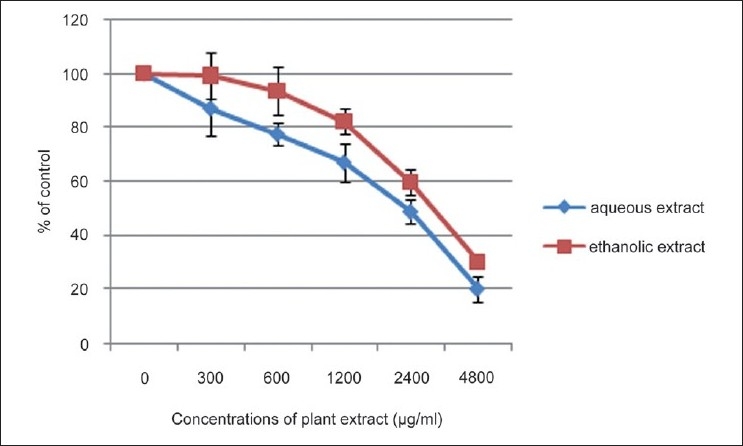
Relative cell survival-SRB assay. Mean± S.D.

Results of the SRB assay are summarized in Figure 3. As evident from this figure, a dose-dependent inhibition of cell survival as assessed by the SRB assay was observed with both aqueous and ethanolic extracts. As was observed in the MTT assay, the inhibition by aqueous extract was significantly (*P* < 0.05) greater than that mediated by the ethanolic extract at all concentrations tested.

## DISCUSSION

Aqueous and ethanolic extracts prepared from a mixture containing equal proportions of *N. sativa* seeds, *H. indicus* roots and *S. glabra* rhizomes were standardized by evaluating different parameters such as organoleptic characters, physicochemical properties, chemical constituents, HPLC, and TLC profiles. The percentage yield of the ethanolic extract and ethanol-extractable matter were greater than the percentage yield of aqueous extract and water-extractable matter. A higher content of total ash, acid-insoluble ash and water-soluble ash were found in the aqueous extract. Our phytochemical screening revealed that both aqueous and ethanolic extracts contained secondary metabolites such as alkaloids, flavanoids, saponins, tannins, steroids, reducing sugars and phenolic compounds. However, higher saponin content was found in the aqueous extract than in the ethanolic extract. HPLC profiles showed the presence of both polar and non-polar compounds in the two extracts. A higher aggregation of polar compounds was seen in the aqueous extract. In all 15 samples (purchased at different times during the year) analyzed by TLC, the same number of spots with the same Rf values were observed. In the HPLC analysis also similar peak profiles was observed in all the samples analyzed. These results show that there was batch to batch consistency of the plant materials used in the preparation of the extracts. The therapeutic value of any drug depends not only on its clinical efficacy, but also in its lack of toxic side-effects. In this investigation, heavy metal analysis showed that the aqueous and ethanolic extracts did not contain detectable levels of Cd, Pb, As and Hg. A previous *in vivo* investigation by Iddamaldeniya *et al*.,[23] has also shown that the aqueous extract (decoction) does not produce any significant toxic effects. These results indicate that the extracts are relatively safe to use for therapeutic purposes.

The results of this study also demonstrated that both the standardized aqueous extract and ethanolic extract prepared from *N. sativa* (seeds), *H. indicus* (roots), and *S. glabra* (rhizome) could exert a strong dose-dependent cytotoxicity to human hepatocellular carcinoma (HepG2) cells *in vitro* as assessed by the inhibitory effects in the MTT and SRB assays. Results with the aqueous extract provide support for the dose-dependent *in vitro* cytotoxic potential of the decoction reported recently by Thabrew *et al*.[10] When comparing the cytotoxic potential of these two extracts, the aqueous extract showed a marginally (though significantly, *P*<0.05) higher activity than the ethanolic extract at all concentrations tested. Thymoquinone is an active compound isolated from *N. sativa* seeds that has been shown to be cytotoxic to several parental and multidrug-resistant human cancer cells.[24,25] HPLC profiles showed that thymoquinone was present in the ethanolic extract but not in the aqueous extract. Thus, compounds other than thymoquinone appear to mediate the cytotoxicity of the standardized aqueous extract of this poly-herbal preparation.

## CONCLUSIONS

Standardization of aqueous and ethanolic extracts of a mixture of *N. sativa* (seeds), *H. indicus* (roots), and *S. glabra* (rhizome) has been carried out according to WHO guidelines. Data obtained may be used as a diagnostic tool for the correct identification of these aqueous or ethanolic extracts. Of the two extracts, the aqueous extract demonstrated marginally greater cytotoxicity despite the absence of thymoquinone in this extract and a higher number of chemical components being extracted into the ethanolic extract as evident from the TLC and HPLC profiles. Results obtained in the present study would be useful for the preparation of a standardized pharmaceutical product that may be used in the future for clinical therapy of hepatocellular carcinoma.
